# A retrospective study of ultrasound-guided intervention for frozen shoulder in the frozen stage

**DOI:** 10.3389/fsurg.2022.998590

**Published:** 2022-10-18

**Authors:** Haitao Guan, Qinfeng Wu, Yuan Zhou, Xing Fan, Kun Zheng, Tong Si, Jinli Zhao

**Affiliations:** ^1^ Department of Ultrasonography, Suzhou Science / Technology Town Hospital, Suzhou, China; ^2^ Department of Ultrasonography, Nantong Third People's Hospital, Nantong, China; ^3^ Department of Rehabilitation, Suzhou Science / Technology Town Hospital, Suzhou, China; ^4^ Department of Pain, Affiliated Hospital of Nantong University, Nantong, China; ^5^ Department of Imaging, Affiliated Hospital of Nantong University, Nantong, China

**Keywords:** frozen shoulder, frozen stage, ultrasound-guided intervention, ultrasound evaluation of the shoulder joint, Constant-Murley score

## Abstract

**Background:**

To investigate the clinical value of ultrasound (US)-guided intervention for frozen shoulder (FS) in the frozen stage.

**Methods:**

This study included 40 patients who had primary FS in the frozen stage and were evaluated by US. These 40 patients have all received conservative treatment elsewhere, and no satisfactory results have been achieved, with no improvement in active and passive movement angles, and no improvement in scores within 3 months. Therefore, their previous treatment was set as comparison. All patients underwent US-guided shoulder joint capsule distension by injection of sterilized water. Of these participants, 22 patients with scapulohumeral periarthritis received a compound betamethasone injection, and 14 patients with thickened coracohumeral ligaments (CHLs) underwent acupotomy lysis, and the remaining 4 patients had no extra treatments. The Constant-Murley score (CMS) was evaluated before and after the operation and analysed for each patient.

**Results:**

Before treatment, the indices for the thickening of the subaxillary joint capsule, subacromial bursa (with or without effusion), long head of the biceps brachii tendon (LHBBT) and CHL were 40, 22, 16 and 14, respectively. After treatment, all the indices were significantly decreased (all *P* < 0.010) except for that of the LHBBT (*P* = 0.123). The patients' CMSs improved, with the median total CMS increasing from 59 points (interquartile range: 53–64 points) to 86 points (interquartile range: 78–90 points) (*P* < 0.010). While the internal rotation (Ir) of the shoulder joint did not improve (FDRs < 0.50), abduction, forward flexion (Ff) and external rotation (Er) improved significantly (all FDRs = 1.00).

**Conclusion:**

Compared with conservative treatment, US-guided intervention for FS in the frozen stage is highly effective and of great clinical value.

## Introduction

Frozen shoulder (FS) is also known as 50-year shoulder, scapulohumeral periarthritis and shoulder contracture syndrome. FS is characterized by pain and is accompanied by shoulder dyskinesia, which gradually worsens. Notably, FS includes three stages: the freezing stage, frozen stage and thawing stage. Patients in the freezing stage can recover on their own after conservative treatment (1–3). However (4, 5), the frozen stage, which is associated with pain and stiffness, can recur and continue for decades (1), which has a significant impact on patients' quality of life, especially middle-aged and elderly patients. However, the treatment of FS in the frozen stage is particularly difficult in the clinical setting. With the widespread application of ultrasound (US) in myology and osteology, US plays an increasingly important role in the diagnosis and treatment of shoulder diseases (6, 7). However, previous studies have emphasized local treatment and ignored the use of holistic treatment (8). Although many patients have no pain after treatment, they are unable to recover their previous exercise ability. In this study, our team performed comprehensive US evaluations of the patients' diseased shoulder joints. On this basis, the FS treatment plan was formulated, and its clinical value was analysed.

## Materials and methods

This retrospective study was approved by the Institutional Ethics Committee of the Nantong third people's hospital. All patients signed an informed consent form before treatment. All of the study protocols adhered to the principles of the Declaration of Helsinki for medical research.

### Patients

From January 2018 to October 2021, FS patients were recruited for this study from Nantong third people's hospital. The inclusion criteria were as follows. (1) The patient had chronic primary FS contracture syndrome and were in the frozen stage. (2) The patient had at least two sets of movements that were limited during active and passive movement, with a range of motion (ROM) of less than 30°. The exclusion criteria were as follows. (1) The patient had metabolic syndrome or diabetes. (2)The patient had an abnormal shoulder bone structure. (3) The patient had a history of shoulder fracture or rotator cuff injury. (4) The patient had secondary FS with other pathogenic factors. (5) The patient had symptoms of muscle atrophy and showed decreased muscle strength. Symptoms and specific scores of patients prior to participation in this study were recorded.

### Procedures

#### US evaluation

All US examinations were conducted by an experienced radiologist (G.H.T., with 10 years of experience) with a US (Arietta 850, Hitachi, Tokyo, Japan) transducer (frequency range: 6–15 MHz). The patient was in a sitting position, and the long head of the biceps brachii tendon (LHBBT), the LHBBT sheath (normal thickness is 1.7 ± 1.6 mm), the subscapular tendon, the supraspinatus tendon, the infraspinatus tendon, the teres minor tendon, the coracohumeral ligament (CHL, 3.08 ± 1.32 mm), the subacromial bursa (0.59 ± 0.17 mm), the rotator interval, the bursae around the rotator cuff, and the subaxillary joint capsule (2.21 ± 0.37 mm) were examined successively according to the US examination method described for the shoulder joint in the Musculoskeletal Ultrasound Technical Guidelines, as recommended by the European Society of Musculoskeletal Radiology (9–12); The dates of all US examinations were recorded along with the results. During the above process, attention was given to observing whether there was pain and dyskinesia caused by an impingement in the shoulder joint cavity or the peripheral capsular ligamentous complex and if there were other pathogenic factors present. The ultrasonographic manifestations of FS include contracture and thickening of the capsule, thickening of the rotator cuff space, thickening of the CHL, and thickening of or effusion from the bursae around the rotator cuff.

#### US-guided intervention treatment

**Figure 1:**
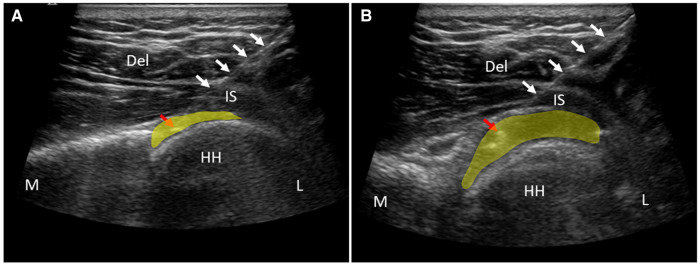
US-guided shoulder joint cavity injection and before (**A**) and after (**B**) distension treatment. Del, deltoid muscle; HH, humeral head; IS, infraspinatus tendon; L, lateral; M, medial. White arrows indicate the injection needle. Red arrow points at the tip of injection needle. The highlighted yellow area indicates the shoulder joint cavity.

**Figure 2:**
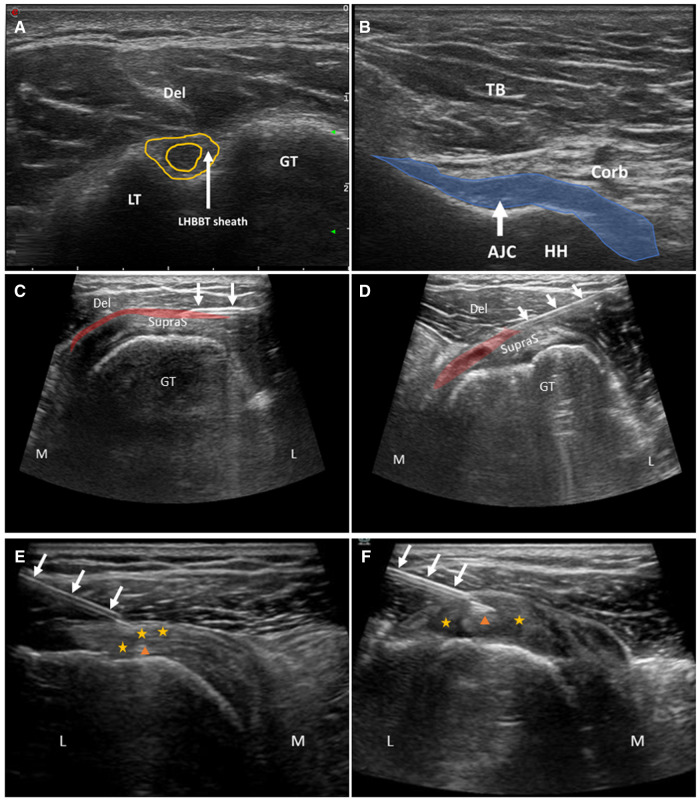
**A,B** are ultrasound images for diagnosis. **C** (before injection) and **D** (after injection) are US-guided injection into the subacromial Bursa. **E** (before injection) and **F** (after injection) are intrathecal injection into the LHBBT sheath. Del, deltoid muscle; TB, Triceps brachii; Corb, coracobrachialis; AJC, axillary joint capsule; HH, humeral head; GT, greater tubercle of humerus; LT, lesser tubercle of humerus; SupraS, supraspinatus tendon; L, lateral; M, medial; yellow star, rotator interval; orange triangle, the long head of the biceps brachii tendon (LHBBT); highlighted red area, subacromial Bursa. White arrows indicate the injection needle.

**Figure 3:**
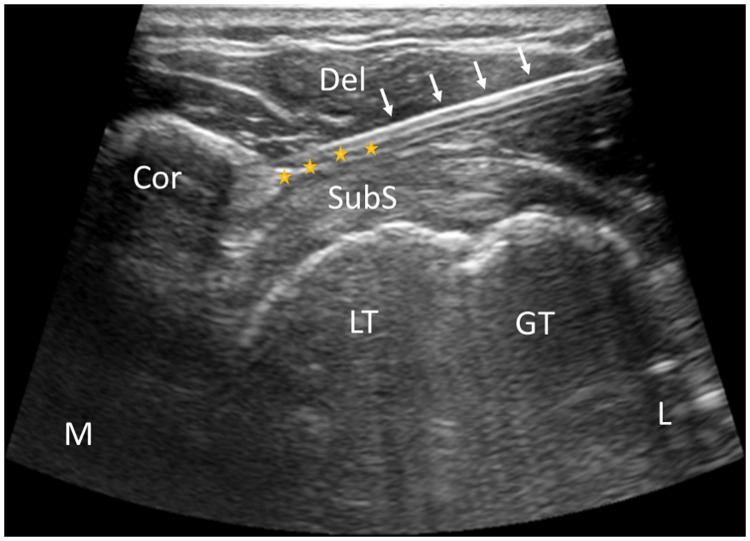
US-guided acupotome lysis of coracohumeral ligaments. Cor, coracoideus; Del, deltoid muscle; GT, greater tubercle of humerus; LT, lesser tubercle of humerus; SubS, subscapularis tendon; L, lateral; M, medial; yellow star, coracohumeral ligament. White arrows indicate the acupotome.

The patients received US-guided intervention treatments during the first, second and fourth weeks (13). After a patient was placed in a lateral recumbent position, the skin was disinfected with povidone iodine, and 0.5% lidocaine was injected for local anaesthesia. Under US guidance, a 22 G injection needle (Kindly, Zhejiang, China) that was connected to a single-use Luer lock syringe (Kindly, Shanghai, China) was inserted into the shoulder joint cavity by a posterior approach. A 10–15 ml volume of sterile water was injected to slowly expand the shoulder joint cavity. When the thickness of the subacromial bursa, the sheath of the LHBBT, or the rotator interval was more than 2 mm, the patient was injected with 1 ml of 10% compound betamethasone. In this manner, the shoulder joint cavity was expanded by water injection under US guidance during the first treatment (Figures 1, 2). Intra-plane injection technique was adopted to conduct the ultrasound-guided subacromial bursa injection. The drug started being slowly pushed out when the bone puncture needle tip reached the subacromial bursa space, and the drug was slowly injected into the subacromial bursa along the injection path from the outside to the inside. The injection was ended until the drug can flow in and out of the subacromial bursa to form a bar-shaped anechoic. When the thickness of the CHL exceeded 3 mm (14), the patient underwent an US-guided acupotomy (diameter: 0.6 mm, ZhongWu, Jiangsu, China) lysis of the CHL during the first treatment. The coracoid process of the CHL was longitudinally thinned and transversely striped by an acupotome three times under sonographic guidance (Figure 3) (15). When a small acupotome was inserted into the coracohumeral ligament under ultrasound guidance, attention should be paid not to damage other structures in the subthoracic space.

#### Shoulder joint Constant-Murley score (CMS)

The shoulder joint CMS provided scores for factors, including pain, daily living activity and the range of motion of the shoulder joint, before and after US-guided treatment (16).

### Statistical analyses

R software (version 4.0.2) was used for the statistical analysis. The normality of the data was tested by the Shapiro–Wilk test. The continuous variables that were normally distributed were expressed as the mean ± standard deviations ($\bar{x}\pm \text{S}$), and the data that were nonnormally distributed were expressed as medians and interquartile ranges (IQR, 25th–75th percentile). Categorical variables are expressed as counts and percentages. The normally distributed data were compared by using the independent samples *t* test. The Mann–Whitney *U* test was used to compare the mean values before and after treatment of the same group or between two groups. The difference in nonnormally distributed variables was compared among multiple groups by using the Kruskal–Wallis test, and the difference between the groups was compared by using the Mann–Whitney *U* test. The nominal *P* value was corrected for multiple comparisons by using the Benjamini–Hochberg method. *P* < 0.050 was considered statistically significant.

## Results

### US evaluation of FS

A total of 40 patients were included in this study; 17 males (42.5%) and 23 females (57.5%), and they had a mean age of 53.0 ± 9.6 years (range, 32–75 years). Before treatment, the median thickness of the subaxillary joint capsule was 3.1 mm (IQR: 2.8–3.8 mm) in 40 patients, and colour Doppler US showed that there was no obvious blood flow signal in the contracted synovium of the capsule. Among them, 22 patients were diagnosed with thickening of the shoulder space with or without effusion, so ultrasound-guided local glucocorticoid injection was added. Fourteen patients were diagnosed with coracohumeral ligament thickening, so ultrasound-guided coracohumeral ligament acupotomy was performed. Four patients were only diagnosed with thickening of the shoulder joint capsule, so only ultrasound-guided injection of sterile water into the shoulder joint cavity was performed. The subacromial bursa (with or without effusion) was thickened in 20 patients, with a median thickness of 2.5 mm (IQR: 2.0–3.5 mm). The LHBBT sheath (with or without effusion) was thickened in 16 patients, and the median thickness of the LHBBT sheath was 6.3 mm (IQR: 2.4–10.0 mm). The median thickness of the CHL was 4.5 mm (IQR: 4.2–4.8 mm) in 14 patients. After treatment, all of the values in the aforementioned indices were significantly lower than the values before treatment (all *P* < 0.010, Table 1), except for the thickness of the LHBBT sheath (with or without effusion) (*P* = 0.123).

**Table 1:** Comparison of the US evaluation results before and after treatment.

	Before treatment	After treatment	*P*
Thickness of the subaxillary joint capsule	3.1 (2.8–3.8)	2.1 (1.5–2.5)	<0.001*
Thickening of the subacromial bursa (with or without effusion)	2.5 (2.0–3.5)	2 (1.9–2.3)	0.009*
Thickness of the LHBBT (with or without effusion)	6.3 (2.4–10.0)	2.5 (2.0–3.8)	0.123
Thickness of the CHL	4.5 (4.2–4.8)	2.5 (2.3–3.4)	0.001*

Data are presented as the median (25th, 75th).

* Statistically significant difference.

### Evaluation on the motion range of the shoulder joint

Before treatment, evaluation of the shoulder joint CMS showed that the median pain score of the patients was 10 points (IQR: 5–10 points), the median score for daily living activity was 11 points (IQR: 7–13 points), and the median score for active range of motion was 16 points (IQR: 12–20 points). After treatment, all aforementioned scores were significantly improved (all *P* < 0.010). Specifically, the median pain score increased to 15 points (IQR: 10–15 points), the median score for daily living activity increased to 18 points (IQR: 16–20 points), and the median score for active range of motion increased to 28 points (IQR: 24–34 points). The median total CMS of the patients increased from 59 points (IQR: 53–64 points) before treatment to 86 points (IQR: 78–90 points) after treatment (*P* < 0.010) (Table 2).

**Table 2:** Comparison of the CMS of the patients before and after treatment.

	Before treatment	After treatment	*P*
Pain (15 points)	10 (5–10)	15 (10–15)	<0.001*
Daily living activity (20 points)	11 (7–13)	18 (16–20)	<0.001*
Activity level (10 points)	5 (3–6)	9.5 (8–10)	<0.001*
Restricted work (4 points)	2 (0–2)	4 (2–4)	<0.001*
Restricted entertainment (4 points)	2 (2–4)	4 (4–4)	<0.001*
Affected sleep (2 points)	1 (0–1)	2 (1–2)	<0.001*
Position reached by painless movement (10 points)	6 (4–8)	8 (8–10)	<0.001*
Angle and score of the active range of motion (40 points)	16 (12–20)	28 (24–34)	<0.001*
Flexion (10 points)	6 (4–8)	10 (8–10)	<0.001*
Abduction (10 points)	4 (2–4)	8 (6–8)	<0.001*
External rotation (10 points)	4 (2–6)	8 (6–8)	<0.001*
Internal rotation (10 points)	2 (2–4)	4 (4–6)	<0.001*
Flexion (angle)	120 (90–123)	170 (150–180)	<0.001*
Abduction (angle)	90 (60–90)	138 (100–153)	<0.001*
Muscle force evaluation (25 points)	25	25	
Total constant score (100 points)	59 (53–64)	86 (78–90)	<0.001*

Data are presented as the median (25th, 75th).

* Statistically significant difference.

**Figure 4:**
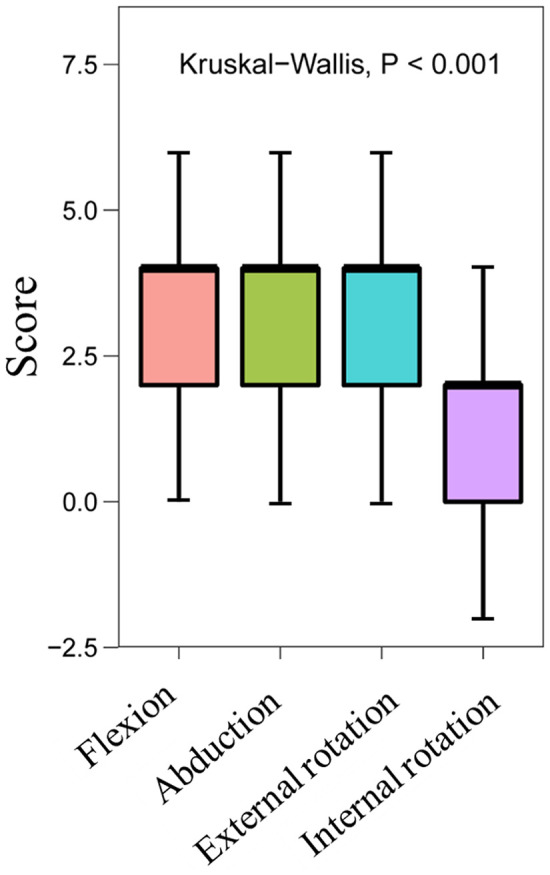
Box chart showing the degree of improvement in the scores of four indices involving the active range of motion.

According to the angle and score of the active ROM in the CMS table, our team found that every subindex (forward flexion (Ff), abduction, external rotation (Er) and internal rotation (Ir)) was significantly improved (*P* < 0.010). The median score for Ff increased from 6 points (IQR: 4–8 points) to 10 points (IQR: 8–10 points), and its median angle increased from 120° (IQR: 90°–123°) to 170° (IQR: 150°–180°). The median score for abduction increased from 4 points (IQR: 2–4 points) to 8 points (IQR: 6–8 points), and its median angle increased from 90° (IQR: 60°–90°) to 138° (IQR: 100°–153°). The median score for Er increased from 4 points (IQR: 2–6 points) to 8 points (IQR: 6–8 points), while the median score for Ir increased from 2 points (IQR: 2–4 points) to 4 points (IQR: 4–6 points) (Table 2). Although there were significant differences in the four index scores that involve the active range of motion of the patients before and after treatment (*P* < 0.010), the degree of improvement of each index was different. It should be emphasized that Ff, abduction and Er showed greater improvement (all FDRs = 1) than Ir (FDRs < 0.050) (Figure 4).

### Comparison of the treatment efficacy of the CHL between the thickening group and the nonthickening group

The patients were divided into the thickening group (*n* = 14) and the nonthickening group (*n* = 26) according to whether the thickening of the patient's CHL was more than 3 mm. In the thickening group, the after-treatment scores of various indices associated with the active range of motion improved compared to the values before treatment (*P* < 0.010). Specifically, the median score for Ff increased from 5 points (IQR: 4–6 points) to 10 points (IQR: 7–10 points), and its median angle increased from 90° (IQR: 80°–120°) to 160° (IQR: 121°–179°). The median score for abduction increased from 2 points (IQR: 2–4 points) to 6 points (IQR: 4–8 points), and its median angle increased from 60° (IQR: 45°–65°) to 110° (IQR: 90°–143°). The median score for Er increased from 3 points (IQR: 2–6 points) to 6 points (IQR: 6–8 points), while the median score for Ir increased from 2 points (IQR: 0–2 points) to 4 points (IQR: 2–4 points). The median total score for the active range of motion increased from 12 points (IQR: 9–18 points) to 24 points (IQR: 20–30 points) (Table 3).

**Table 3:** Comparison of the active range of motion in the thickening group before and after treatment.

	Before treatment	After treatment	*P*
Angle and score of the active range of motion	12 (9–18)	24 (20–30)	0.001*
Flexion (10 points)	5 (4–6)	10 (7–10)	<0.001*
Abduction (10 points)	2 (2–4)	6 (4–8)	<0.001*
External rotation (10 points)	3 (2–6)	6 (6–8)	0.001*
Internal rotation (10 points)	2 (0–2)	4 (2–4)	0.004*
Flexion (angle)	90 (80–120)	160 (121–179)	0.002*
Abduction (angle)	60 (45–65)	110 (90–143)	0.002*

Data are presented as the median (25th, 75th).

* Statistically significant difference.

In the nonthickening group, the median score for Ff increased from 6 points (IQR: 5–8 points) to 10 points (IQR: 8–10 points), and its median angle increased from 120° (IQR: 90°–146°) to 175° (IQR: 150°–180°). The median score for abduction increased from 4 points (IQR: 4–6 points) to 8 points (IQR: 6–8 points), and its median angle increased from 90° (IQR: 65°–90°) to 150° (IQR: 120°–158°). The median score for Er increased from 4 points (IQR: 2–6 points) to 8 points (IQR: 6–8 points), while the median score for Ir increased from 2 points (IQR: 2–4 points) to 4 points (IQR: 4–6 points). The median total score for the active range of motion increased from 17 points (IQR: 15–24 points) to 29 points (IQR: 26–34 points) (Table 4).

**Table 4:** Comparison of the active range of motion in the nonthickening group before and after treatment.

	Before treatment	After treatment	*P*
Angle and score of the active range of motion (*n* = 40)	17 (15–24)	29 (26–34)	<0.001*
Flexion (10 points)	6 (5–8)	10 (8–10)	<0.001*
Abduction (10 points)	4 (4–6)	8 (6–8)	<0.001*
External rotation (10 points)	4 (2–6)	8 (6–8)	<0.001*
Internal rotation (10 points)	2 (2–4)	4 (4–6)	<0.001*
Flexion (angle)	120 (90–146)	175 (150–180)	<0.001*
Abduction (angle)	90 (65–90)	150 (120–158)	<0.001*

Data are presented as the median (25th, 75th).

* Statistically significant difference.

After treatment, there was no significant difference in the improvement of the active range of activity between the thickening group and the nonthickening group (all *P* > 0.050) (Table 5).

**Table 5:** Comparison of the improvements in the active range of motion between the thickening group and the nonthickening group before and after treatment.

	Thickening group	Nonthickening group	*P*
Angle and score of the active range of motion (*n* = 40)	12 (10–16)	12 (8–14)	0.404
Flexion (10 points)	4 (4–4)	4 (2–4)	0.188
Abduction (10 points)	4 (2–4)	4 (2–4)	0.867
External rotation (10 points)	2 (2–4)	4 (2–4)	0.672
Internal rotation (10 points)	2 (1–2)	2 (0–2)	0.618
Flexion (angle)	60 (45–60)	55 (30–61)	0.718
Abduction (angle)	50 (35–75)	55 (30–64)	0.917

Data are presented as the median (25th, 75th).

## Discussion

The pathogenesis of FS is unclear. It may be related to continuous tension in the patient's posture and the patient's emotions, or related to an increase in a variety of inflammatory factors (17, 18). The disease includes the freezing stage, frozen stage and thawing stage (5). Although FS is self-limiting, some patients may experience the frozen stage for decades, and the condition can recur. In this study, our team focused on patients with FS in the frozen stage. There are various treatments for FS (16, 19–22), such as acupuncture, massage, release of the shoulder joint cavity under brachial plexus block, arthroscopic release of the shoulder joint capsule, and high-dose oral steroids. However, the efficacy of these treatments is uncertain. Lu et al. (23) found that US-guided distension of the shoulder joint cavity had good efficacy for the treatment of FS. However, in the frozen stage, CHL thickness correlates negatively with the Er and Ir ranges of the shoulder (14), and FS patients with thickening of the CHL do not achieve good improvement of shoulder ROM using this method alone (24). Many studies have shown that steroid drugs can effectively control pain associated with FS (25), but these drugs are relatively short acting (26, 27). Traditional or single US-guided injection therapy is not the best method to treat FS, especially for patients in the frozen stage. Preoperative US evaluation is very important. The ultimate aim of US-guided intervention should be to improve the overall function of the shoulder joint.

During the active and passive movement of the shoulder joint, US can dynamically determine whether the sliding trajectory and the structures around the tender points are abnormal, and these evaluations cannot be obtained by magnetic resonance imaging or other imaging examinations. The US manifestations of FS include contracture and thickening of the subaxillary joint capsule, thickening of the coracobrachial tendon, coracoacromial ligament and scapulohumeral periarthritis (28, 29). US-guided interventional treatment is increasingly applied as an FS therapy and has shown a good curative effect. Several studies have shown that the range of motion of the shoulder joint in patients with primary FS is significantly improved after an US-guided shoulder joint injection (30, 31). However, there are few reports on the application of US-guided treatment for FS patients in the frozen stage.

In terms of improving the shoulder joint function, the short- and medium-term efficacy of an US-guided shoulder joint injection combined with joint capsule distension is significantly better than that of a simple shoulder joint injection (23, 32, 33). A study by Cheng et al. (23, 34) showed that three consecutive US-guided shoulder joint injections combined with joint capsule distension was an effective method for the treatment of FS. Therefore, in this study, our team also used a series of three consecutive US-guided shoulder joint injections combined with joint capsule distension. Ultrasound images showed subacromial bursitis when the thickness of the synovium and/or effusion is greater than 2 mm. It can be diagnosed as long head biceps tenosynovitis or inflammation of the rotator cuff space when local thickening and hypoechoic reduction and the long head of the biceps brachii tendon sheath or rotator cuff space, and the thickness of the synovium and/or effusion is greater than 2 mm. Therefore, we believe that there is inflammation when the thickness of the peri-shoulder space is greater than 2 mm, and local glucocorticoid injection under ultrasound guidance is performed (35). The normal volume of the shoulder joint cavity is approximately 20 ml, but in patients with shoulder contracture syndrome, the volume is often reduced due to intra-articular synovial contracture. In this study, 10–15 ml sterilized water was injected into the shoulder joint cavity. The injection dose that is used depends on the patient's tolerance level. In patients with FS, fibrosis and thickening of the capsule were the main features that were seen in the shoulder joint cavity. Colour Doppler US showed that there was no obvious blood flow signal in the contracted synovium of the shoulder capsule, which means there was no evidence of inflammation in the capsule. A study showed that shoulder joint capsule distension with sterilized water is effective for release training (33). The expansion of the shoulder joint cavity by injecting sterile water is mainly to mechanically separate the adhering joint cavity and increase the range of motion, which was proved a good long-term effect. Therefore, only sterilized water was used for the joint injections in this study.

Inflammation, indicated by signs such as effusion and reduced tendon clearance space, can be identified when an US evaluation is performed to evaluate the soft tissue around the shoulder joint and the rotator cuff tendon clearance. Several studies have shown that synovitis can be effectively controlled by steroids (26, 27). Therefore, US-guided steroid injections into the soft tissue around the shoulder joint and rotator cuff tendon clearance may also be effective. However, the curative efficacy of these US-guided local injections is variable. This may be because the compound betamethasone injection is a lipid-soluble granular suspension. This limits the drug from being applied evenly on the synovial membranes or in the interstitial spaces with adhesions due to the lack of flow, which prevents the drug from reaching some places. Therefore, to solve the above problems, our team shook it well before the injection. During the injection, the needle was inserted into the gap or synovium, and the drug was slowly injected, with mild pressure being applied when any resistance was encountered. The injection was stopped when an arc-shaped anechoic region appeared in the injection area (36).

The CHL is often found to be thickened in FS patients. When the CHL is thickened up to 3.0 mm, there is a high likelihood for the existence of FS. The thickening of the CHL is highly correlated with adhesions, inflammation, and stability of the shoulder joint (14). The CHL is stiffer in the frozen stage than in the freezing stage (24). Some studies have shown that US-guided release of the CHL is effective in FS patients (37, 38). Under US guidance, our team made vertical cuts in the CHL at the coracoid process with a small needle knife in 14 patients with thickened CHLs; this procedure achieved a good curative effect.

In this study, US-guided FS intervention treatment was performed based on static and dynamic US evaluations, and a holistic treatment was emphasized. The CMS of the shoulder joint, especially the daily living activity score, was significantly improved after the operation. The Ff, abduction and Er of the shoulder joint were significantly improved, but the Ir was not improved. The reason may be that Ir is a compound action, and the patients' symptoms improved slowly due to severe adhesions and fibrosis of the shoulder joint during the frozen stage. Two patients were found severe fibrosis of the shoulder joint cavity during the operations. Besides, this study ignored the assessment of the patient's posture, but rounded shoulder posture is one of the most common structural abnormalities of the shoulder complex, involving increased cervical anteversion and increased upper thoracic retroversion, resulting in shoulder and scapula herniation, as well as increase in inferior rotation and anterior tilt. Patients with frozen shoulder typically have a rounded shoulder with adducted and internally rotated glenohumeral joints, and a contracture of the joint capsule (39, 40). After the expansion of the shoulder joint, the joint capsule contracture improved and the range of motion increased, but the patient's rounded shoulder did not change. When the adduction and internal rotation test was performed, the subacromial rotation and anteversion of the scapula were limited, and the subacromial space was insufficient, which could cause pain and limitation (41). Therefore, it is suggested that the patients with frozen shoulder still need to adjust their posture through manipulation and training after the release of the joint capsule and related ligaments in order to comprehensively improve the shoulder joint function.

In conclusion, US-guided intervention of FS patients in the frozen stage is convenient and effective; this procedure has high application value and should be promoted in clinical practice.

## Limitations

The internal rotation is affected by the coracobrachialis muscle, infraspinatus muscle, and teres minor muscle. For the thickening and nonthickening group, the internal rotation of CMS did not improve, Potential approaches to operate the muscles or further improve this score need be explored in the future. Severe adhesions and fibrosis of the shoulder joint might be one of the reasons, and this need be further studied.

## Data Availability

The raw data supporting the conclusions of this article will be made available by the authors, without undue reservation.
